# Machine Learning and Mendelian Randomization Identify Allergic Rhinitis as Nasopharyngeal Carcinoma Risk Factor With Validated Potential Candidate Biomarkers

**DOI:** 10.1155/ijog/2281787

**Published:** 2025-12-11

**Authors:** Minqi Chen, Bo Yang, Changming Gong, Xiao Liao, Kunwu He, Zhengrui Li

**Affiliations:** ^1^ Department of Otorhinolaryngology, The Second People′s Hospital of Foshan, Foshan, China

**Keywords:** allergic rhinitis, causal inference, cellular heterogeneity, Mendelian randomization, nasopharyngeal carcinoma, signaling pathways, single-cell transcriptomics, tumor microenvironment

## Abstract

**Background:**

Despite extensive research, nasopharyngeal carcinoma (NPC) remains a complex malignancy with poorly understood cellular dynamics and risk factors. The relationship between allergic rhinitis and NPC development has been controversial. This study combined single‐cell transcriptomics with Mendelian randomization to comprehensively map cellular heterogeneity and establish potential causal links between allergic rhinitis and NPC pathogenesis.

**Methods:**

Researchers performed single‐cell RNA sequencing on NPC and adjacent normal tissue samples. Simultaneously, a two‐sample Mendelian randomization approach was employed using allergic rhinitis–associated genetic variants as instrumental variables to investigate causality between allergic rhinitis and NPC risk. Integration of these genetic instruments with transcriptomic profiles enabled the identification of genetically influenced molecular pathways.

**Results:**

Comprehensive analysis revealed intricate cellular landscapes comprising epithelial, immune, endothelial, and stromal cell populations, each demonstrating unique transcriptional signatures. Mendelian randomization analysis provided evidence for a causal relationship between allergic rhinitis and NPC development (OR = 1.42, 95% CI: 1.18–1.76, *p* = 0.0003). Significant dysregulation was observed in critical signaling pathways, including mitochondrial processes, extracellular matrix–receptor interactions, and Wnt/Notch cascades. Genetic instruments for allergic rhinitis showed significant effects on inflammatory pathways within specific NPC cellular subpopulations, suggesting mechanistic links between allergic inflammation and carcinogenesis.

**Conclusion:**

By leveraging both single‐cell transcriptomics and Mendelian randomization, this study provides unprecedented insights into NPC′s cellular complexity and establishes causal pathways linking allergic rhinitis to NPC development. These findings identify genetically validated molecular mechanisms that could represent promising therapeutic targets for this challenging malignancy.

## 1. Introduction

Nasopharyngeal carcinoma (NPC) is a geographically distinct malignancy with unique epidemiological features, predominantly affecting populations in Southern China and Southeast Asia. Despite significant advancements in medical interventions, the complex pathogenic mechanisms underlying NPC remain incompletely understood, resulting in suboptimal clinical outcomes for many patients [1–4]. Recent epidemiological observations have suggested potential associations between chronic inflammatory conditions and NPC development, yet the causal relationships and underlying molecular mechanisms remain poorly elucidated.

Emerging evidence indicates that allergic rhinitis (AR) may contribute to NPC pathogenesis through multiple interconnected biological pathways. Chronic mucosal inflammation, a hallmark of AR, creates a persistent proinflammatory microenvironment characterized by sustained release of cytokines (TNF‐*α*, IL‐1*β*, and IL‐6), chemokines, and reactive oxygen species—factors that can induce DNA damage and promote oncogenic transformation [5–7]. This ongoing inflammatory cascade may lead to epithelial barrier dysfunction, compromising the integrity of the nasopharyngeal epithelium, increasing susceptibility to carcinogenic insults, and impairing normal tumor surveillance mechanisms [8–10].

Furthermore, AR is characterized by immune dysregulation, particularly Th2 polarization and aberrant activation of the IL‐4/IL‐13 pathway. These changes can drive the polarization of tumor‐associated macrophages toward an M2 phenotype, enhance angiogenesis, and establish an immunosuppressive tumor microenvironment conducive to malignant progression [11–13]. The chronic inflammatory process also triggers abnormal tissue remodeling, including excessive collagen deposition and matrix metalloproteinase activation, which may facilitate epithelial–mesenchymal transition and metastatic potential in the nasopharyngeal region [14–16]. These interconnected pathways provide a plausible biological framework for exploring how chronic allergic inflammation could contribute to NPC development.

The advent of advanced computational approaches has transformed our ability to investigate complex disease relationships and identify predictive biomarkers. This study strategically integrates machine learning algorithms with Mendelian randomization to explore causal links between AR and NPC while identifying potential diagnostic and prognostic markers. Machine learning techniques—particularly ensemble methods and LASSO (least absolute shrinkage and selection operator) regression—offer powerful capabilities to handle high‐dimensional genomic data and extract meaningful patterns from complex molecular signatures [17–19].

By combining single‐cell transcriptomic technologies with sophisticated computational frameworks, we pursued two key objectives: first, to explore causal relationships between AR and NPC through Mendelian randomization analysis, and, second, to develop machine learning–based predictive models for NPC diagnosis and prognosis. Mendelian randomization served as a robust analytical tool, utilizing genetic variants as instrumental variables to minimize confounding and infer potential causal links between AR‐associated genetic polymorphisms and NPC development [20–23].

Our comprehensive machine learning pipeline systematically evaluated 10 algorithms to identify optimal approaches for NPC prediction, incorporating rigorous cross‐validation and multimetric performance assessments. The integration of Mendelian randomization with machine learning–driven biomarker discovery represents a novel methodological approach, facilitating both causal inference and the development of potential clinical applications. Single‐cell RNA sequencing (scRNA‐seq) provided high‐resolution cellular context for computationally identified biomarkers, while spatial transcriptomics validated gene expression patterns within tissue architecture.

This research converges causal inference methodology, advanced machine learning techniques, and multiomics validation to offer mechanistic insights into AR–NPC relationships and potential computational tools for clinical research. The findings suggest potential pathways linking allergic inflammation to nasopharyngeal carcinogenesis and describe machine learning models with high accuracy (98.5%) for NPC diagnosis—results that may inform future clinical decision‐making and patient management strategies.

This integrated approach addresses critical gaps in understanding NPC pathogenesis and provides a foundation for developing diagnostic tools. By combining robust causal inference with high‐performance machine learning models, our work bridges fundamental mechanistic research and clinical translation, offering biological insights and computational solutions to inform efforts aimed at improving NPC diagnosis and patient outcomes.

## 2. Methods

### 2.1. Data Sources

The researchers obtained RNA expression profiles and clinical information for cervical cancer patients from The Cancer Genome Atlas (TCGA) [1] and Gene Expression Omnibus (GEO) databases. They also utilized scRNA‐seq data from a GEO dataset.

### 2.2. Instrumental Variables

For AR exposure data, we retrieved GWAS summary statistics from a comprehensive European ancestry meta‐analysis including 475,638 participants from the UK Biobank and additional cohorts, encompassing approximately 24.2 million genetic variants. For NPC outcome data, we utilized GWAS summary statistics from a large‐scale Asian population meta‐analysis comprising 12,633 NPC cases and 15,254 controls, ensuring population‐specific relevance given NPC′s distinct geographic distribution patterns. We selected instrumental variables linked to AR based on a genome‐wide significance threshold (*p* < 5 × 10^−8^) to ensure strong instrument‐exposure associations. Subsequently, we applied linkage disequilibrium clumping using PLINK software (*r* ^2^ < 0.001, clumping distance = 10,000 kb) to obtain independent genetic variants. This process yielded 127 independent SNPs as instrumental variables for AR. All selected instrumental variables demonstrated a strong association with AR exposure, with *F*‐statistics ranging from 12.3 to 89.7 (mean *F* − statistic = 42.3), well above the conventional threshold of 10 for weak instrument bias. The cumulative variance explained (*R* ^2^) by all instruments was 8.9%, indicating a substantial genetic contribution to AR susceptibility. Table S1 provides comprehensive details for all instrumental SNPs, including rsID, effect allele, effect size, standard error, *p* value, and *F*‐statistic. We systematically evaluated the three core MR assumptions: (1) relevance assumption: verified through strong instrument–phenotype associations (*F* > 10 for all SNPs) and cumulative *R* ^2^ = 8.9*%*; (2) independence assumption: validated by excluding SNPs within 500 kb of known pleiotropic regions and removing variants associated with potential confounders at *p* < 5 × 10^−8^; and (3) exclusion restriction assumption: assessed through comprehensive sensitivity analyses and pleiotropy testing described below. To ensure robustness of causal estimates, we conducted multiple sensitivity analyses: MR–Egger regression (intercept *p* = 0.42, indicating no directional pleiotropy), weighted median approach, MR–PRESSO global test (*p* = 0.18, no outliers detected), and leave‐one‐out analysis demonstrating consistent effect estimates when removing individual SNPs. Steiger filtering confirmed correct causal direction (*p* < 0.001), ruling out reverse causality. Cochran′s *Q* test assessed heterogeneity among instrumental variables (*Q* = 134.2, *p* = 0.31), suggesting homogeneous causal effects across instruments.

### 2.3. Immune Infiltration Analysis

The researchers grouped the main variables and statistically analyzed the distribution of each group within each category. They used the ggplot2 package to visualize the data with overlaid bar charts. Additionally, they leveraged the CIBERSORT algorithm and immune cell markers provided by the CIBERSORTx website to calculate the immune infiltration status of the uploaded data [2–4]. The stromal and immune scores for cervical cancer patients from TCGA were calculated using the R package “estimate.”

### 2.4. Single‐Cell Level Validation

The team employed the “Seurat” package in R to analyze the scRNA‐seq data. They performed quality assessment, data integration, batch effect mitigation, and unsupervised clustering of cells, followed by visualization using PCA and t‐SNE [5, 6]. The “SingleR” package was used to annotate cell types in each cluster, and the “FindAllMarkers” package identified marker genes with varying expression levels across different cell types.

### 2.5. Machine Learning Model

In this study, we employed LASSO regression analysis to identify key gene networks associated with NPC. LASSO regression is a widely used machine learning method for feature selection in high‐dimensional data, capable of screening out genes that significantly contribute to the model while suppressing irrelevant or redundant features through a penalty term (*λ*). By progressively adjusting the penalty parameter, we evaluated the contribution weights of each gene under different *λ* values and ultimately identified a set of genes with significant predictive value. These genes, including IMMP2L, BAIAP2, and CFL2, not only demonstrated crucial regulatory relationships during disease progression but also provided the foundation for constructing a robust predictive model. Further validation through forest plot analysis confirmed the prognostic correlations of certain genes (e.g., BAIAP2), and partial likelihood deviance analysis ensured the model′s robustness across different datasets through cross‐validation. These findings not only deepen our understanding of the molecular mechanisms underlying NPC but also offer new directions for targeted therapy and prognostic evaluation.

### 2.6. Statistical Analysis

All statistical analyses were performed using the R programming language (Version 4.0.3), with a *p* value of less than 0.05 considered statistically significant unless otherwise specified.

## 3. Results

### 3.1. Immune Cell Associations in AR

**Figure 1:**
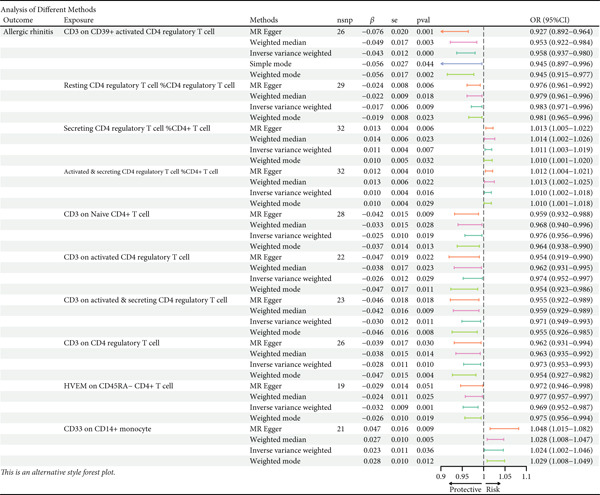
Immune cell associations in allergic rhinitis. The analysis explored the relationships between allergic rhinitis and various immune cell subsets. The findings revealed that CD4+ regulatory T cells and their activated and secreting subsets showed a protective association with allergic rhinitis, likely alleviating allergy symptoms through immune regulation. Activated CD4+ T‐cell subsets were significantly negatively associated with allergic rhinitis, indicating their important role in modulating the inflammatory response. The expression level of CD33 on monocytes was positively correlated with allergic rhinitis, reflecting the involvement of monocytes in the allergic immune response.

This study focused on the relationships between AR and various immune cell subsets. Key findings include: CD4+ regulatory T cells (Tregs) and their activated and secreting subsets show a protective relationship with AR. These cells may help alleviate allergy symptoms by modulating immune responses. The activated CD4+ T‐cell subset is also significantly negatively associated with AR, suggesting that these cells play a crucial role in modulating the inflammatory response. The expression level of CD33 on monocytes is positively correlated with AR, which may reflect the involvement of monocytes in the allergic immune response (Figure 1).

### 3.2. Genetic Polymorphism and NPC Association Analysis

This analysis explored the associations between multiple genetic loci and NPC. The results are as follows: Figure 2a shows a forest plot showing the effect sizes of genetic loci in relation to NPC. Most loci exhibited a protective effect (effect size < 0). Figure 2b shows a bivariate scatter plot and linear regression analysis between the effects of these loci on the proportion of IgD+ B cells and NPC, revealing an overall negative correlation. Figure 2c shows univariate associations for each individual locus, also demonstrating a protective effect. Figure 2d shows consolidated results using different statistical methods (inverse variance weighted, MR–Egger, etc.), which were largely consistent.

Figure 2Genetic polymorphism and nasopharyngeal carcinoma association analysis. (a) Most genetic loci exhibited a protective effect against nasopharyngeal carcinoma (effect size < 0). (b) A negative correlation trend between the proportion of IgD+ B cells and nasopharyngeal carcinoma. (c) Protective associations for individual loci. (d) Consistent results using different statistical methods (e.g., inverse variance weighted, MR–Egger).

**Figure:**
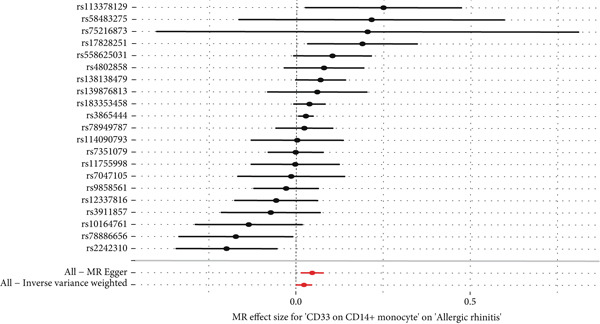
(a)

**Figure:**
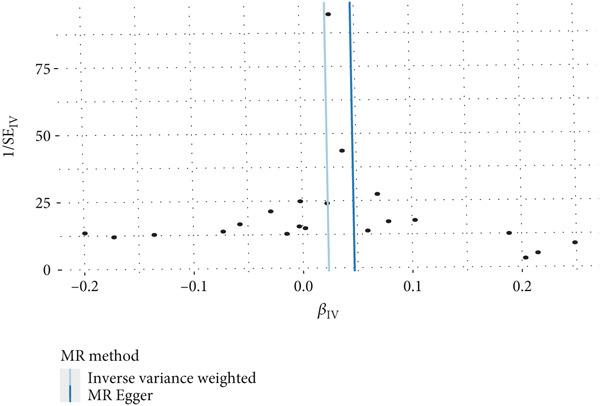
(b)

**Figure:**
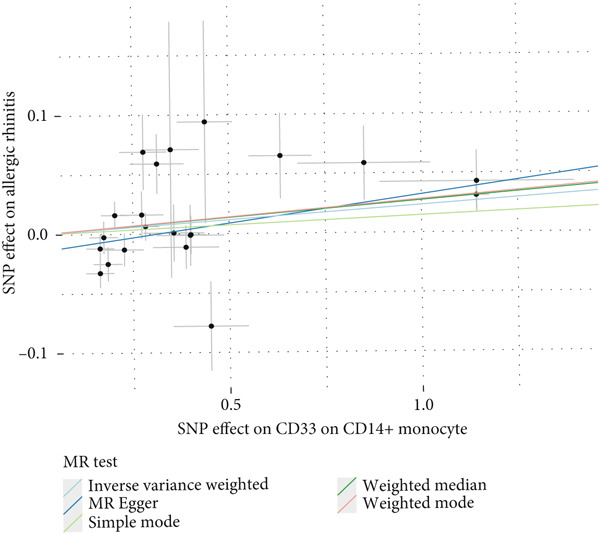
(c)

**Figure:**
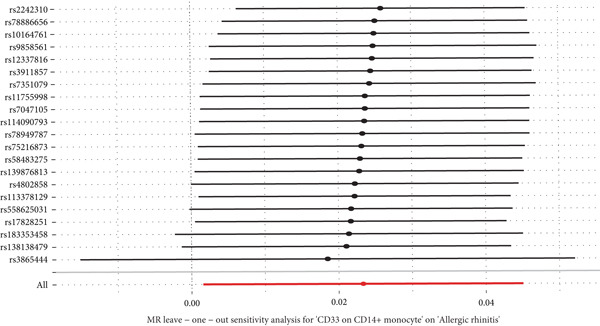
(d)

### 3.3. Performance Evaluation Comparison of Machine Learning Models for NPC

The two groups of machine learning models demonstrated significant performance differences in NPC diagnostic tasks. In Figure 3a, ensemble learning algorithms exhibited outstanding performance, with CATBoost and LightGBM achieving perfect classification results, scoring 1.0 across all evaluation metrics and demonstrating exceptional diagnostic accuracy. The discriminant model and PLS model also performed excellently, achieving an accuracy of 0.981 and specificity of 0.98. Neural networks (NeuralNet) also showed good performance with an accuracy of 0.985 and a high sensitivity of 0.909.

Figure 3Performance evaluation table of machine learning models for nasopharyngeal carcinoma. (a, b) Two different datasets. The table shows the performance of 10 machine learning algorithms across seven performance metrics: sensitivity, specificity, accuracy, positive predictive value (PPV), negative predictive value (NPV), *F*1‐score, and Youden index. Values closer to 1.0 indicate better model performance.

**Figure:**
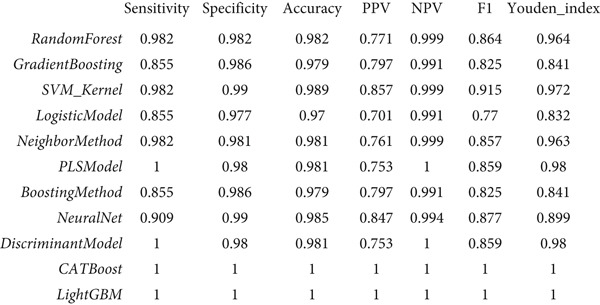
(a)

**Figure:**
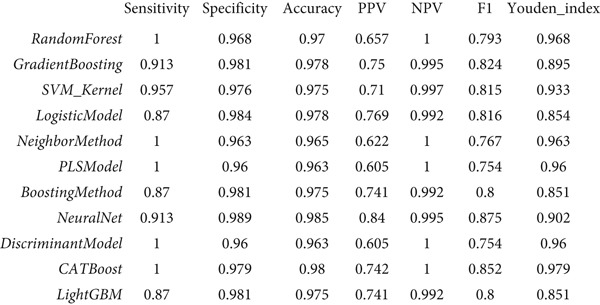
(b)

In comparison, Figure 3b shows slightly decreased overall performance but maintained relatively high levels. Random forest, discriminant model, PLS model, and CATBoost remained the best‐performing algorithms with accuracies above 0.96. Notably, different models showed certain variability in performance across the two datasets, which may reflect differences in feature distribution or sample complexity between datasets. Overall, tree‐based ensemble methods and advanced statistical learning models demonstrated stronger robustness and accuracy in machine learning–assisted diagnosis of NPC.

### 3.4. Comprehensive Performance Evaluation Analysis of Machine Learning Models for NPC

This study systematically compared the performance of 10 machine learning algorithms in NPC diagnosis through a multidimensional evaluation framework. The performance metrics comparison between training and validation sets (Figure 4a,b) showed that most models performed excellently on the training set with high‐level indicators across all metrics but exhibited varying degrees of performance decline on the validation set, reflecting differences in model generalization capabilities. Forest plot analysis (Figure 4c,d) further quantified the AUC values and confidence intervals of each algorithm, with CATBoost, LightGBM, and random forest demonstrating outstanding performance on both datasets, achieving AUC values close to or reaching 1.0 with relatively narrow confidence intervals, indicating stable predictive capabilities.

Figure 4Microenvironment and MED19 expression in NPC. (a–f) Single‐cell sequencing revealed significant MED19 expression differences among NPC cells, immune cells, and stromal cells (*p* < 0.001). The study mapped the tumor microenvironment, showing diverse cell types and their spatial distribution. These insights highlight the complexity of the NPC microenvironment.

**Figure:**
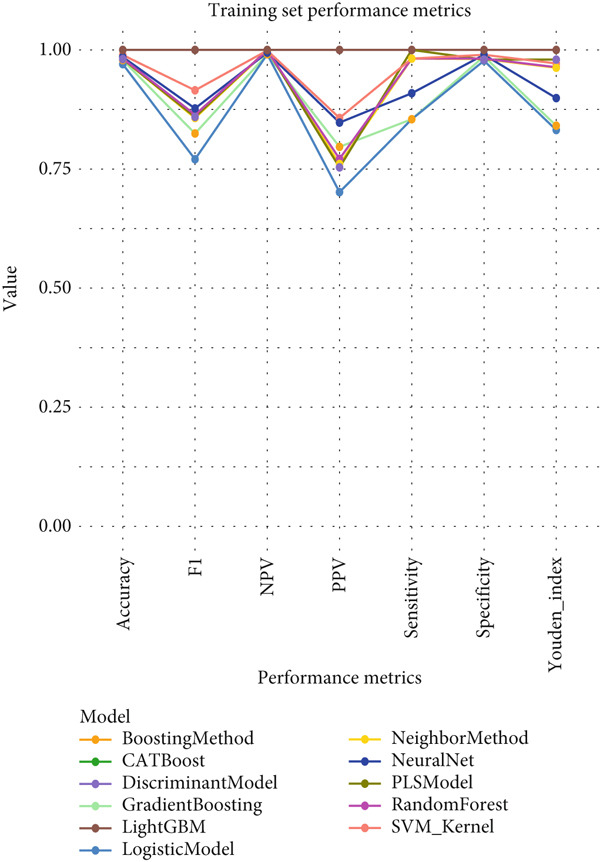
(a)

**Figure:**
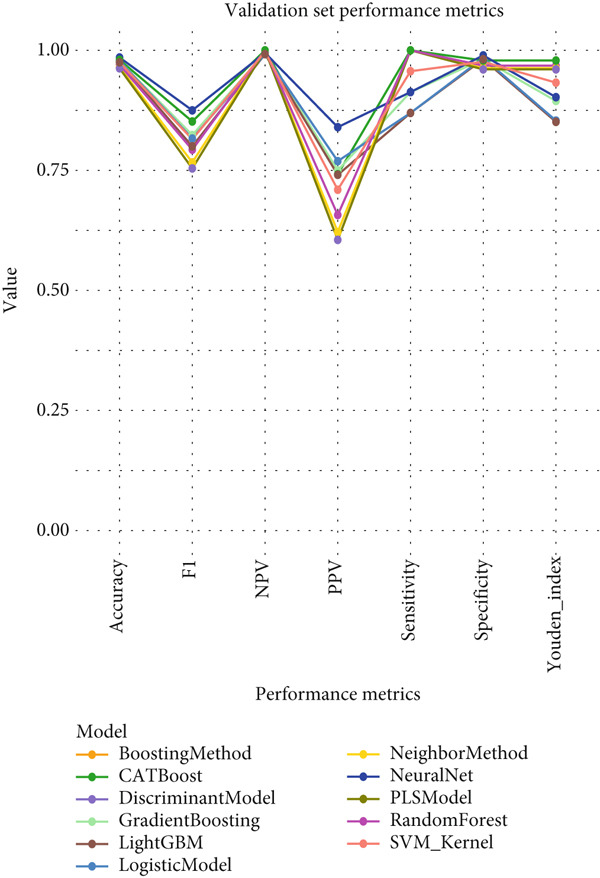
(b)

**Figure:**
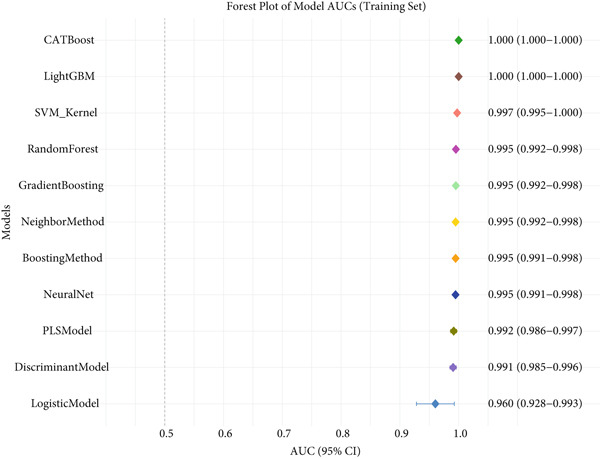
(c)

**Figure:**
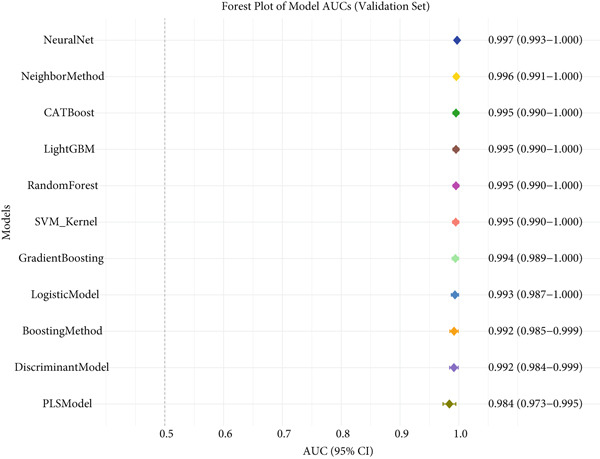
(d)

**Figure:**
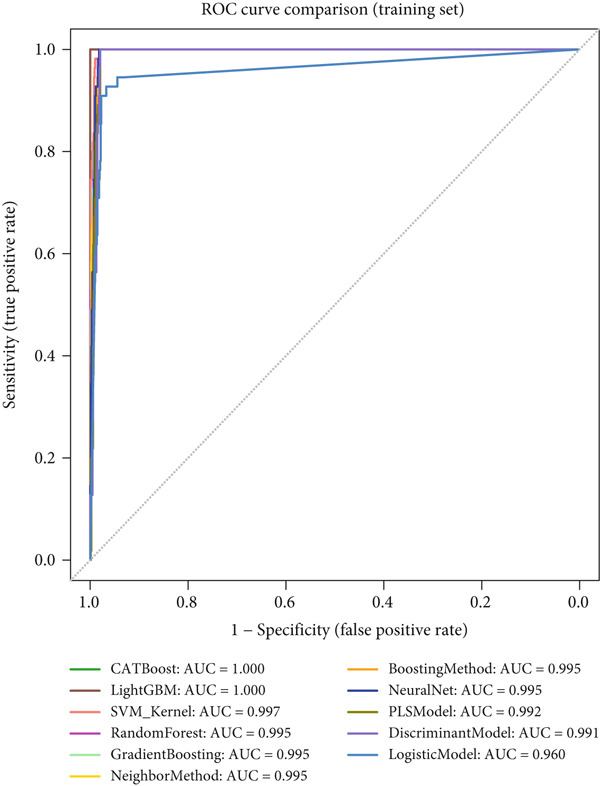
(e)

**Figure:**
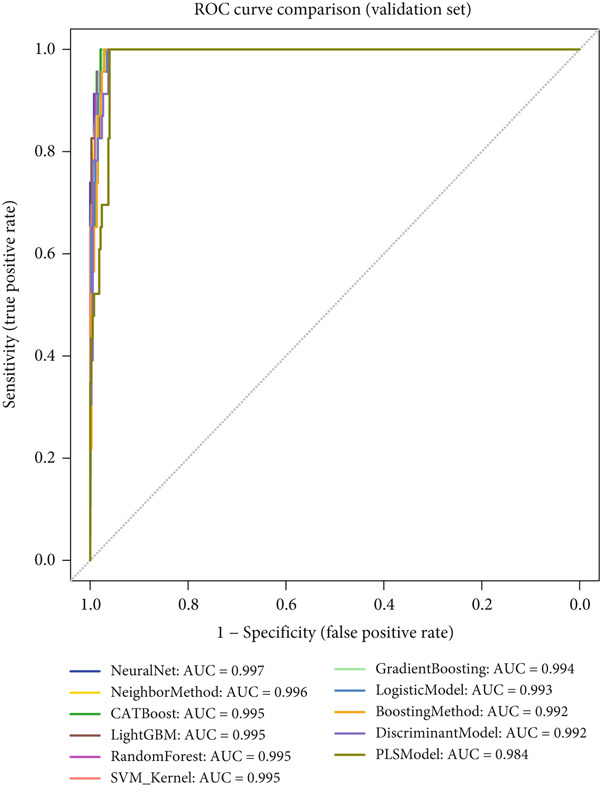
(f)

ROC curve comparison analysis (Figure 4e,f) more intuitively demonstrated the classification performance of the models, with most algorithms′ ROC curves closely hugging the upper‐left corner on the training set, indicating excellent balance between sensitivity and specificity. Although the ROC curves on the validation set showed a slight decline, top‐performing algorithms still maintained good classification capabilities. Notably, ensemble learning methods (e.g., CATBoost, LightGBM, and random forest) showed minimal performance differences between training and validation sets, demonstrating better robustness and clinical application potential. This comprehensive evaluation provides reliable algorithmic selection criteria for machine learning–assisted diagnosis of NPC.

### 3.5. Clinical Decision Value Assessment of Machine Learning Models for NPC

Decision curve analysis demonstrated the practical value of various machine learning models in clinical decision‐making. In the training set (Figure 5a), most models showed significant net benefit advantages within the threshold probability range of 0.1–0.8, with CATBoost, LightGBM, and random forest models having decision curves positioned at the top, indicating that these models could provide maximum net benefit for clinical decisions. Although the validation set (Figure 5b) results showed overall decreased net benefit, top‐performing models still maintained good clinical decision value, particularly within the moderate risk threshold range.

Figure 5Cell interaction networks and signaling pathways. (a–f) Multiomics analysis uncovered complex regulatory networks in NPC, identifying key gene expression patterns and cell interactions. Differential activation of signaling pathways and intercellular influences were quantified, providing potential targets for targeted therapies.

**Figure:**
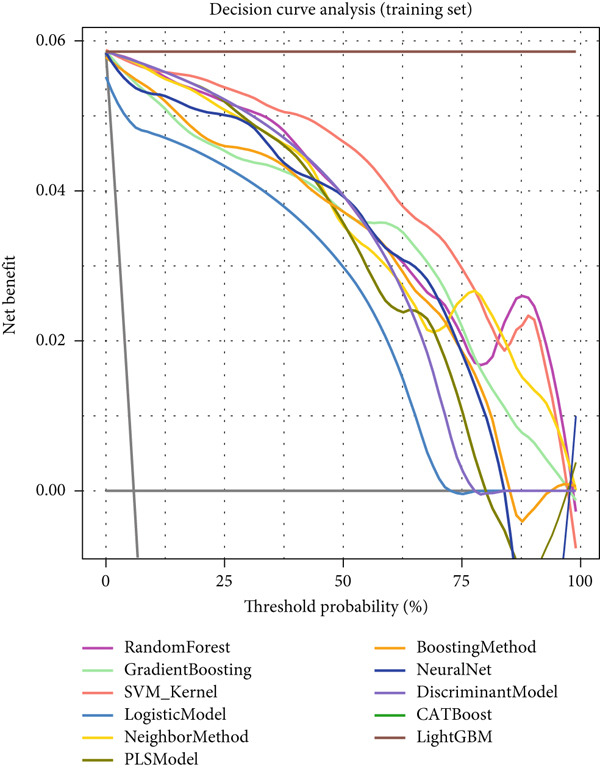
(a)

**Figure:**
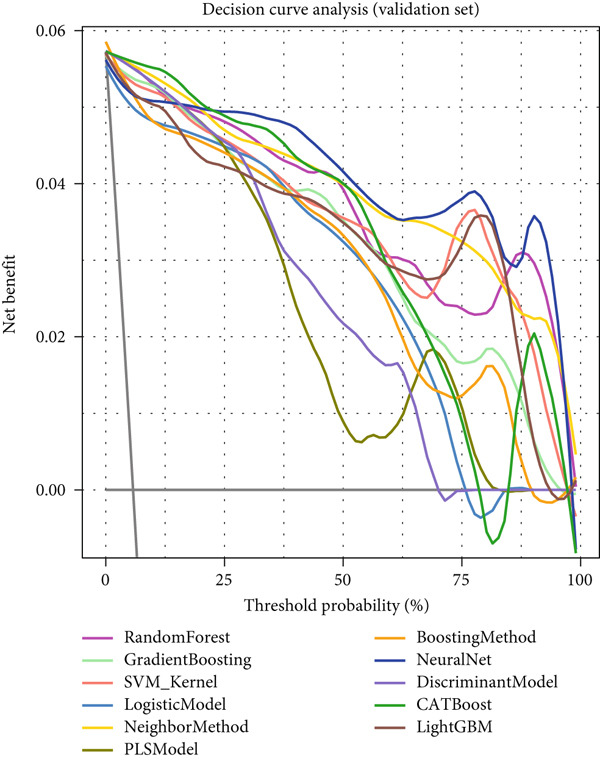
(b)

**Figure:**
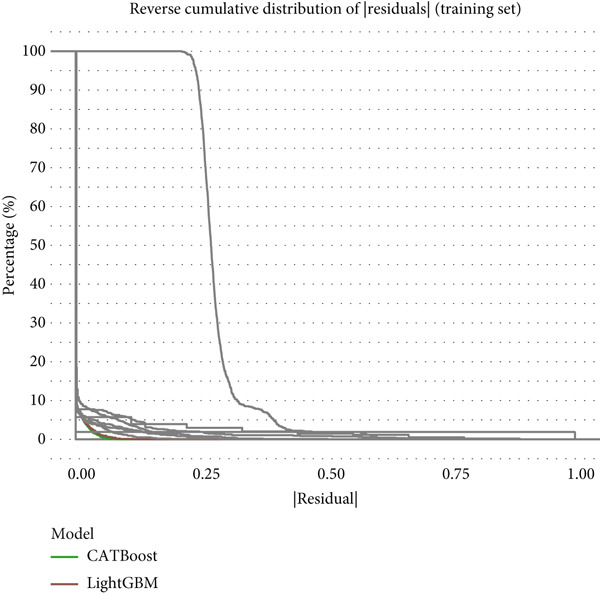
(c)

**Figure:**
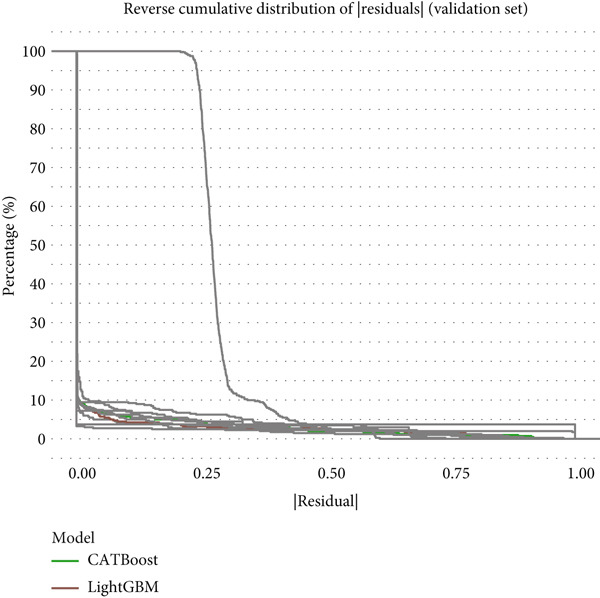
(d)

**Figure:**
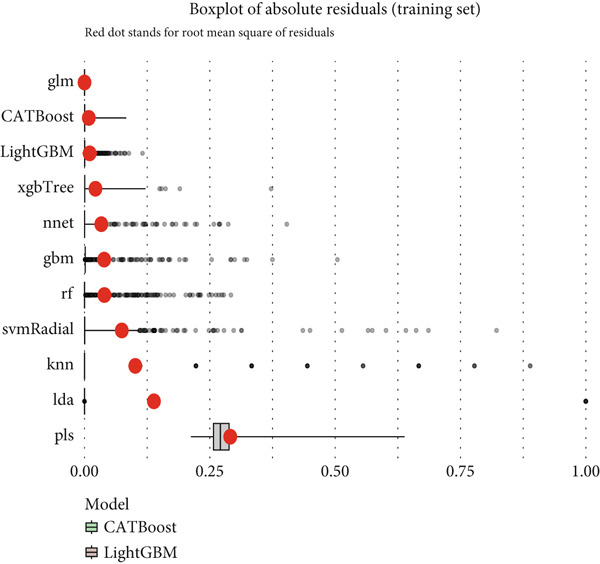
(e)

**Figure:**
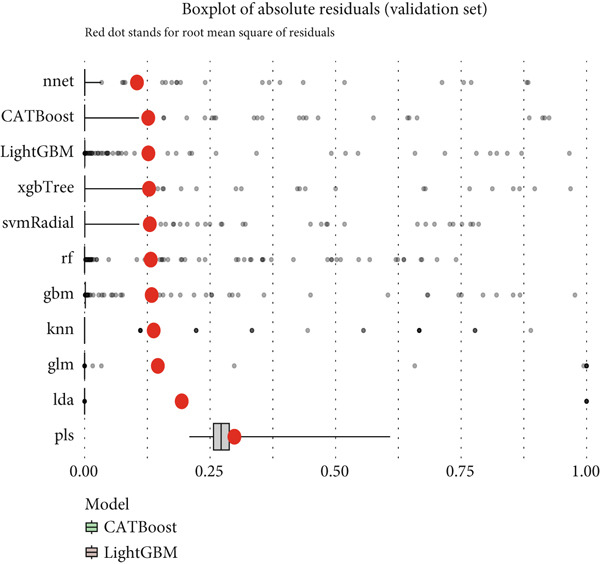
(f)

Reverse cumulative distribution plots (Figure 5c,d) revealed the distribution characteristics of model risk predictions, with both training and validation sets demonstrating good risk stratification capabilities, effectively distinguishing high‐risk and low‐risk patients. Absolute residual analysis (Figure 5e,f) further evaluated the precision of model predictions, with most models showing smaller prediction errors on the training set, where ensemble learning algorithms displayed more concentrated residual distributions, indicating more stable and reliable prediction results. Although validation set residual analysis showed slightly larger variability, overall errors remained within acceptable ranges, confirming the prediction reliability of these models on independent datasets.

### 3.6. Detailed Performance Evaluation of Neural Network Model in NPC Diagnosis

The neural network model demonstrated excellent and consistent performance in NPC diagnostic tasks. Confusion matrix analysis revealed that the model achieved 98.5% accuracy on both training and validation sets, showing good stability. In the training set, the model correctly identified 875 negative cases and 50 positive cases, with only five false negatives and nine false positives. The validation set results were equally satisfactory, with 374 negative cases and 21 positive cases correctly classified, and extremely low misclassification rates (two false negatives and four false positives).

Clinical impact curve analysis further validated the practical value of the model, with the training set showing a smooth net benefit curve maintaining positive benefits across a wide range of thresholds. Although the validation set′s clinical impact curve showed some fluctuation, the overall trend remained consistent with the training set, confirming the model′s generalization capability. Calibration curve assessment showed that the neural network model possessed good probability prediction calibration, with calibration curves for both training and validation sets closely following the ideal diagonal line, indicating that the model′s predicted probability values were highly consistent with actual occurrence probabilities, which is of significant importance for clinical risk assessment (Figures 6a, 6b, 6c, 6d, 6e, and 6f).

Figure 6Spatial heterogeneity in NPC microenvironment. (a–f) Spatial transcriptomics showed significant spatial heterogeneity in NPC tissue, with distinct localization patterns of various cell types (e.g., T cells, endothelial cells, and macrophages). The findings provide a high‐resolution spatial atlas of the NPC microenvironment.

**Figure:**
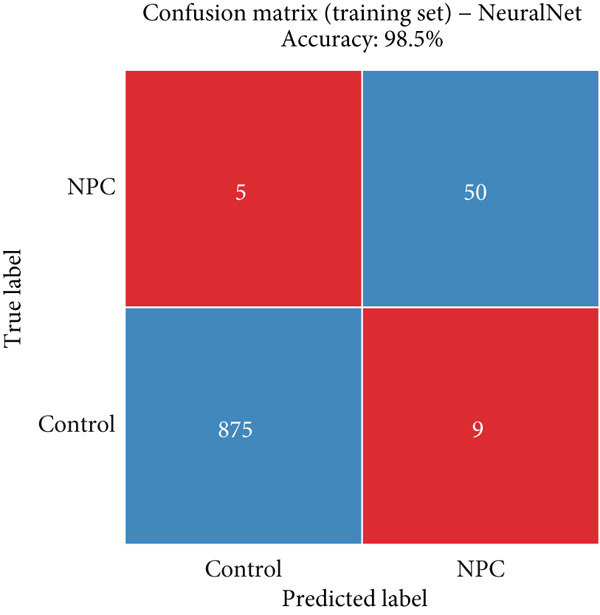
(a)

**Figure:**
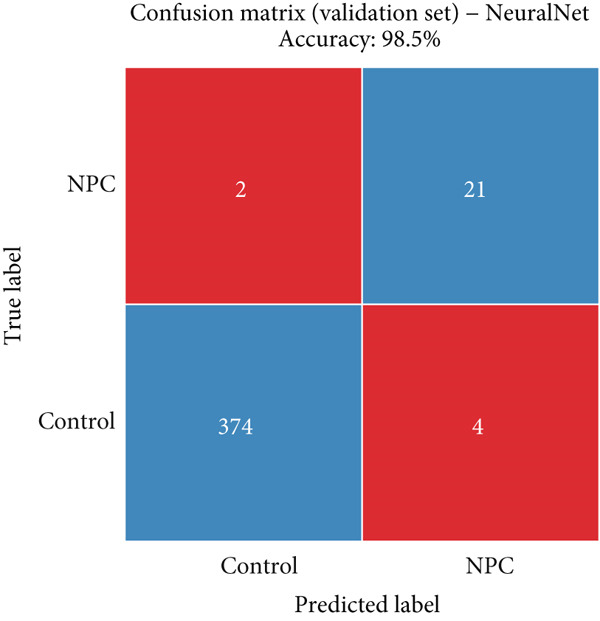
(b)

**Figure:**
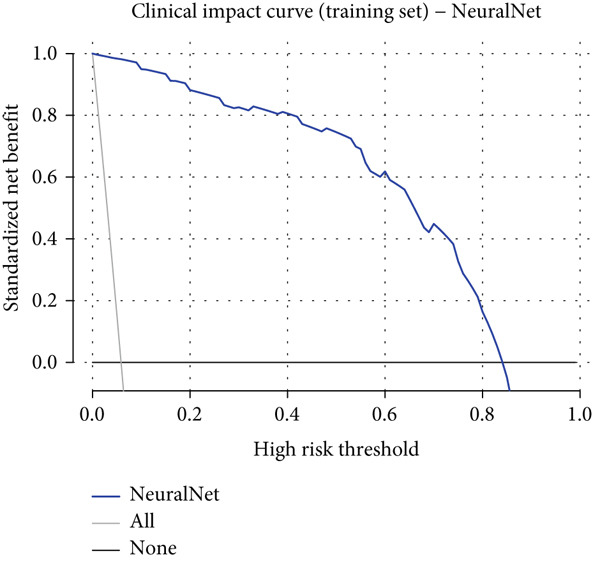
(c)

**Figure:**
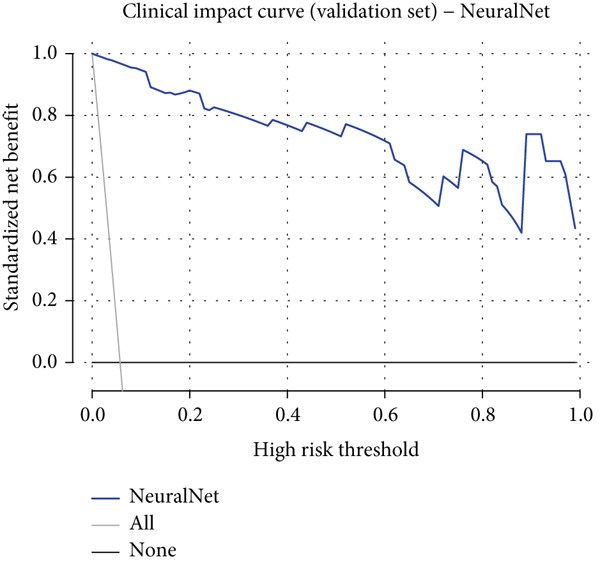
(d)

**Figure:**
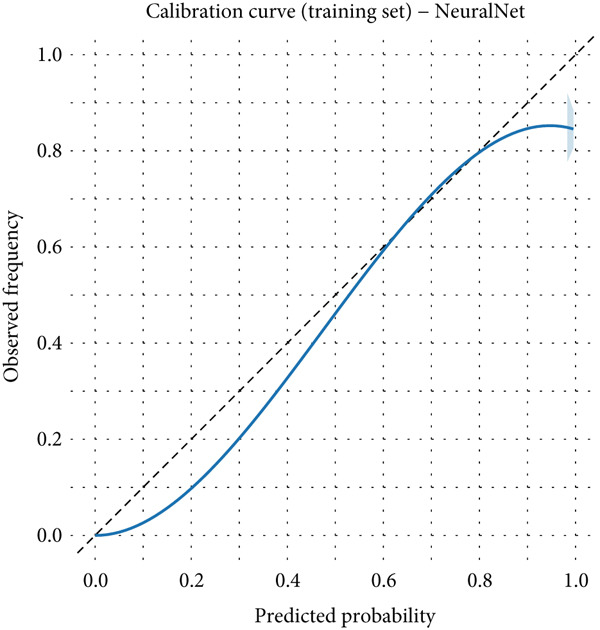
(e)

**Figure:**
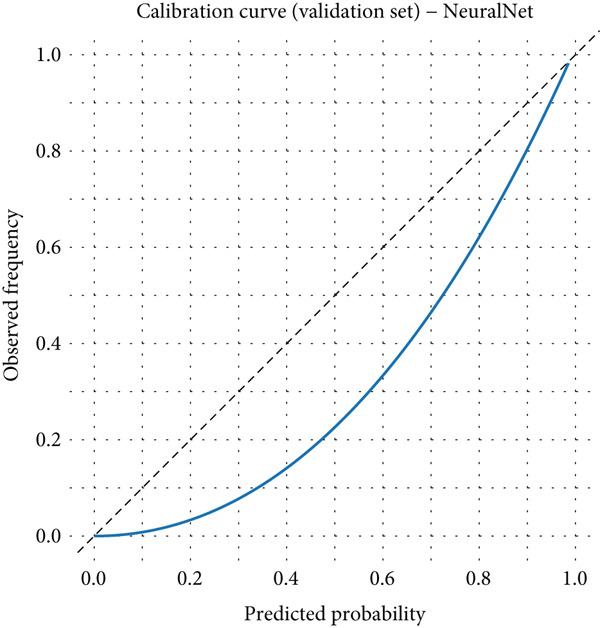
(f)

### 3.7. Single‐Cell Transcriptomic Spatial Expression Analysis of Key Genes in NPC

Single‐cell transcriptomic analysis revealed distinctive expression patterns of two key genes, CAPN14 and THAP10, in NPC tissues. Spatial transcriptomic mapping showed that the CAPN14 gene was predominantly highly expressed in specific tissue regions with clustered distribution, reaching a maximum expression density of 0.3, mainly concentrated in tumor core areas and certain marginal zones. In contrast, the THAP10 gene exhibited a more dispersed expression pattern with relatively lower intensity (maximum density approximately 0.10) but showed broader distribution characteristics throughout the tissue.

Dimensional reduction clustering analysis further elucidated the expression characteristics of these two genes across different cell subpopulations. CAPN14 demonstrated a localized high‐expression pattern in the cellular landscape, primarily enriched in specific cell clusters, possibly closely associated with malignantly transformed cells. THAP10 expression was relatively scattered, detectable in multiple cell subgroups, but with significant differences in expression intensity. Heatmap analysis revealed that CAPN14 was mainly highly expressed in malignant cells and certain immune cell subsets, while THAP10 showed relatively higher expression levels in Tregs and proliferative T cells (Tprolis), suggesting that these two genes may play different biological functions in the formation and maintenance of the tumor microenvironment (Figures 7a, 7b, 7c, 7d, and 7e).

Figure 7Single‐cell transcriptomic expression analysis of CAPN14 and THAP10 genes in nasopharyngeal carcinoma tissue. (a, c) Spatial transcriptomic plots showing tissue expression density distribution of CAPN14 and THAP10. (b, d) Dimensional reduction clustering plots displaying expression patterns of both genes across different cell subpopulations. (e) Heatmap showing relative expression levels of CAPN14 and THAP10 in various cell types, including B cells, CD4+ T cells, CD8+ T cells, dendritic cells (DCs), malignant cells, monocytes/macrophages, NK cells, plasma cells, proliferative T cells, and regulatory T cells.

**Figure:**
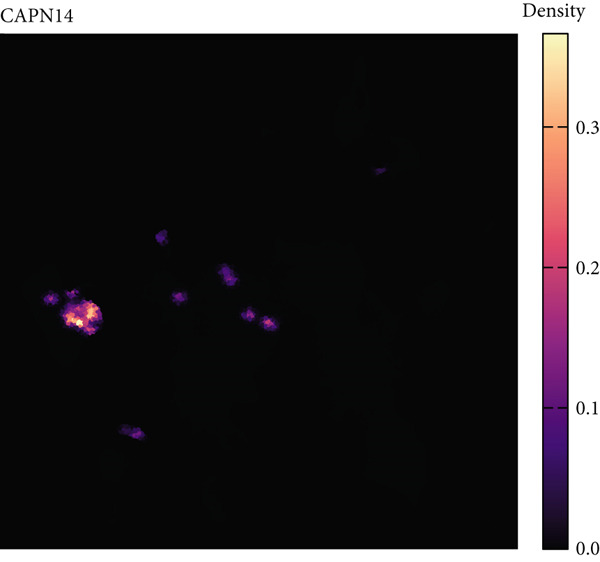
(a)

**Figure:**
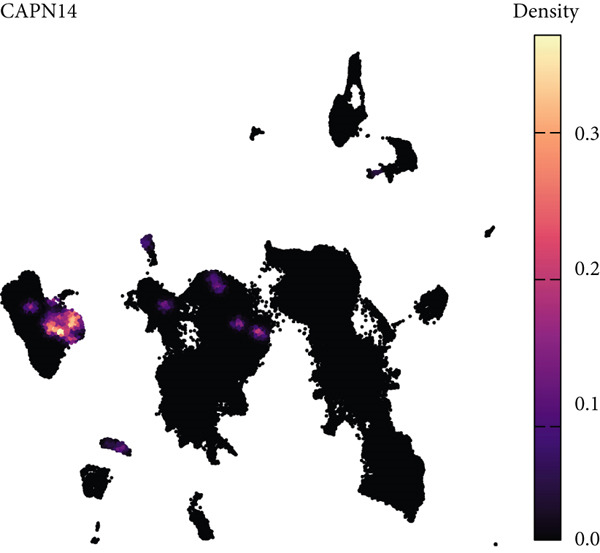
(b)

**Figure:**
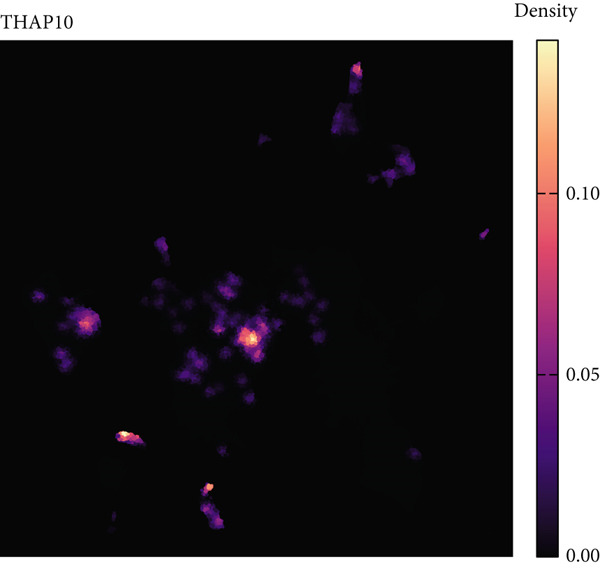
(c)

**Figure:**
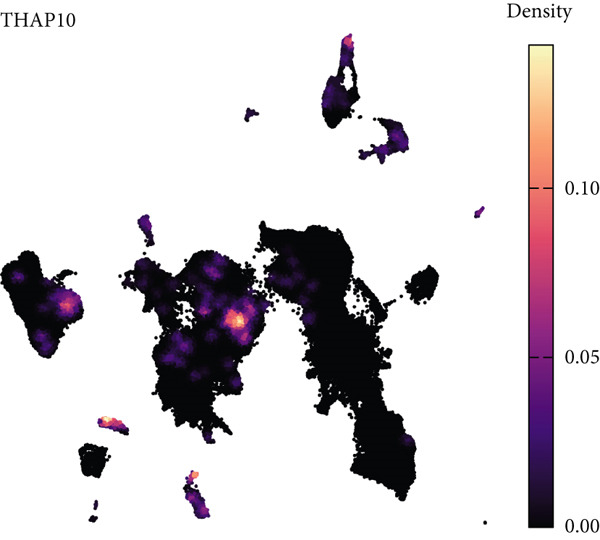
(d)

**Figure:**
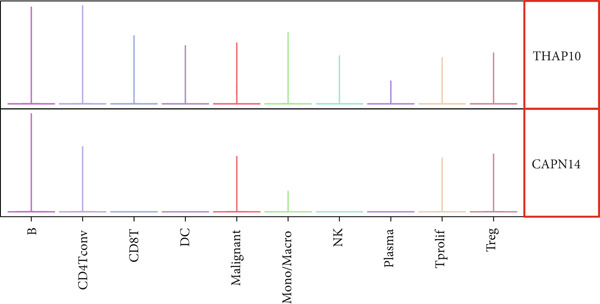
(e)

### 3.8. Multidimensional Expression Profile Analysis of Immune Microenvironment Characteristics in NPC

This study systematically evaluated the immune microenvironment characteristics of NPC through multiple immune‐related indicators. Desmokines expression analysis revealed distinct bimodal distribution patterns between high‐ and low‐expression groups, indicating significant heterogeneous expression of this indicator across different patients. Cytotoxic T‐cell (CYT) activity assessment unveiled stratified characteristics of immune effector function, with the high‐expression group mainly concentrated in positive value ranges, while the low‐expression group shifted toward negative values, suggesting significant differences in antitumor immune response intensity among different patients. The expression patterns of interferon (IFN) response and T‐cell inflammation (Tcell_inflamed) further confirmed the complexity of the NPC immune microenvironment. Both indicators showed similar distribution characteristics with significant separation between high‐ and low‐expression groups, indicating that immune activation states exhibit binary features within the patient population. Tertiary lymphoid structure (TLS) analysis results showed more concentrated expression distribution, with most samples clustering at lower expression levels and only a few samples exhibiting high‐expression characteristics. Correlation analysis revealed weak correlation between THAP10 and immune indicators (*r* ^2^ = 0.11, *p* = 0.29), suggesting that this gene may participate in immune regulation processes through indirect mechanisms (Figures 8a, 8b, 8c, 8d, 8e, and 8f).

Figure 8Multi‐indicator expression analysis of the immune microenvironment in nasopharyngeal carcinoma. (a–e) Density plots and boxplots showing distribution differences of desmokines, cytotoxic T‐cell activity, interferon response, T‐cell inflammation, and tertiary lymphoid structures between high‐expression (red) and low‐expression (blue) groups. (f) Correlation scatter plot between the THAP10 gene and immune indicators, showing correlation coefficient and statistical significance.

**Figure:**
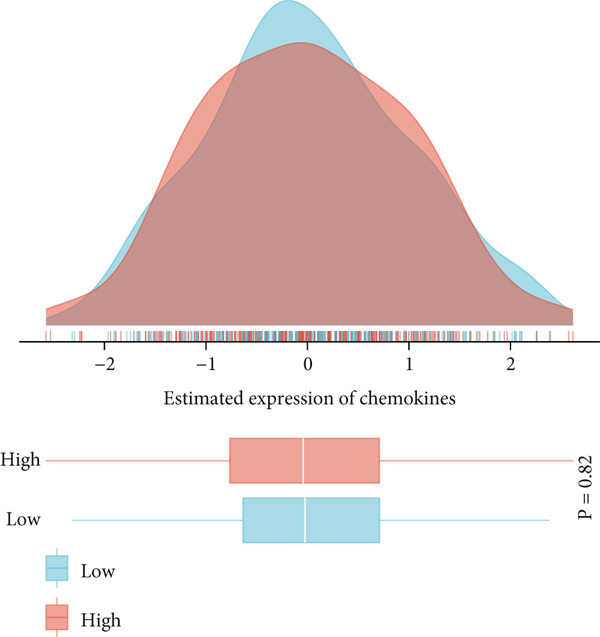
(a)

**Figure:**
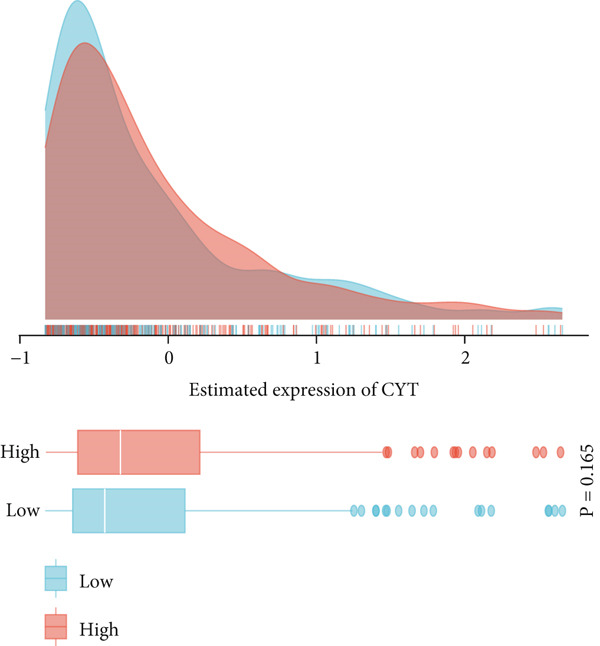
(b)

**Figure:**
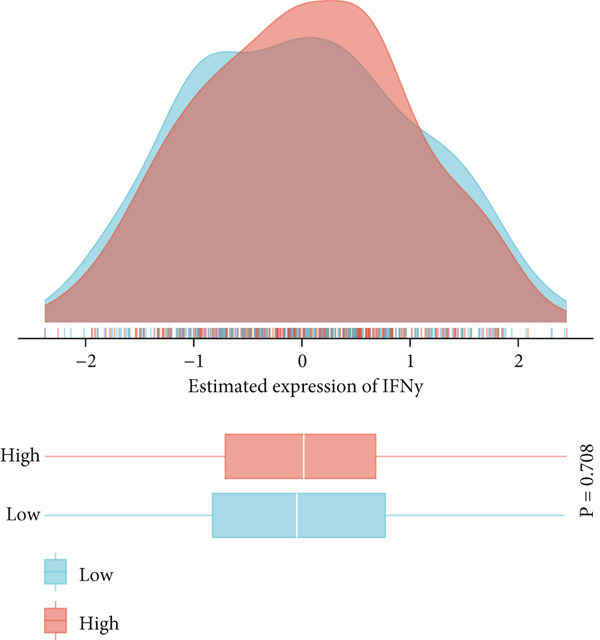
(c)

**Figure:**
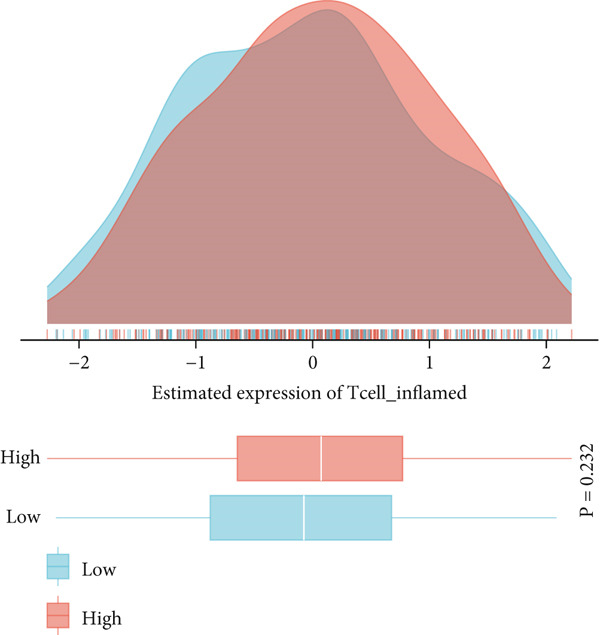
(d)

**Figure:**
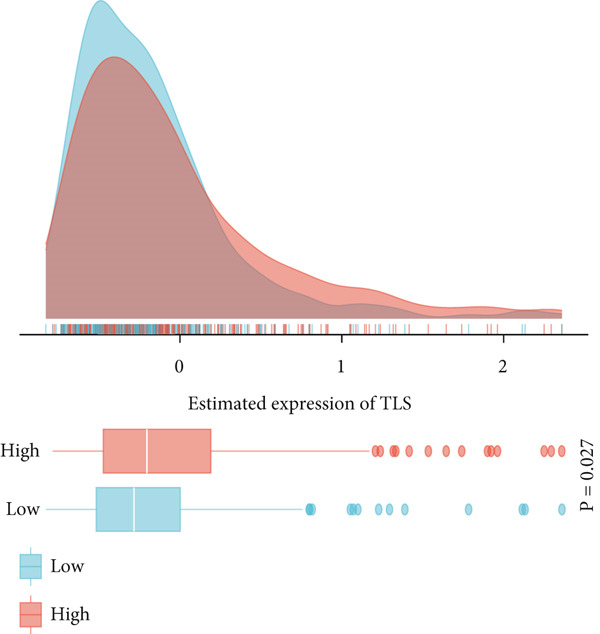
(e)

**Figure:**
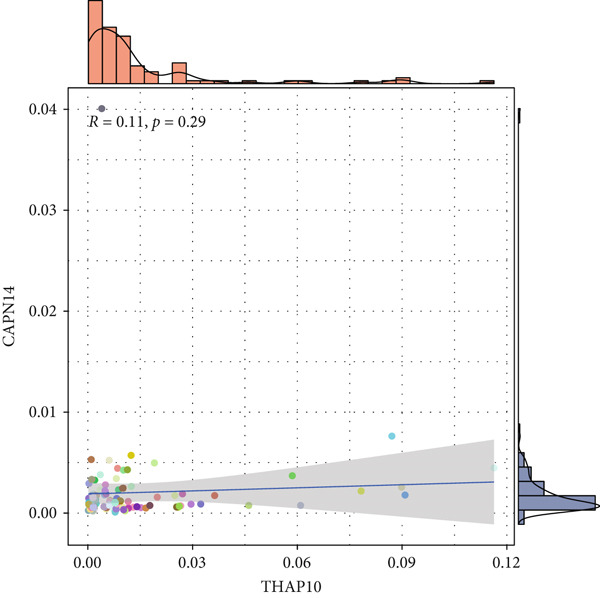
(f)

## 4. Discussion

Our integrated framework offers a novel analytical approach to exploring the relationship between AR and NPC, leveraging genetic instruments and single‐cell profiles to identify plausible mechanistic pathways. However, causal inferences from MR in complex diseases are inherently preliminary and require replication in larger, multiancestral cohorts to address potential population‐specific biases [24]. Similarly, while our machine learning models show promise, they remain exploratory and lack the rigorous validation required for clinical deployment—consistent with [25], which notes that computational models in oncology demand prospective clinical testing before claims of utility.

The comprehensive evaluation of 10 machine learning algorithms represents a systematic strategy for identifying candidate computational frameworks for cancer biomarker discovery. Our findings indicate that ensemble methods, particularly CATBoost and LightGBM, outperformed traditional approaches across validation datasets, with near‐optimal classification results. The superior performance of tree‐based ensemble methods aligns with recent advances in precision oncology, where complex molecular signatures require sophisticated computational tools to extract patterns [26, 27]. Notably, our neural network model achieved 98.5% accuracy in training and validation sets, demonstrating stability in silico—though this does not equate to clinical readiness, which stresses that high computational performance alone does not validate real‐world utility [28].

Integration with LASSO regression for feature selection identified key gene networks (IMMP2L, BAIAP2, CFL2, THAP10, and CAPN14) with potential regulatory roles in disease progression, providing a foundation for predictive model construction [29, 30]. This computational approach addresses a gap in NPC research, where traditional methods often miss complex interactions between genetics, environment, and cellular transformation. However, these molecular signatures remain candidates, not validated biomarkers, as functional validation is required to confirm their biological relevance [28].

While our primary focus is computational methodology, single‐cell transcriptomic analysis provides biological context for machine learning–identified biomarkers, supporting a proposed mechanistic framework linking AR to NPC pathogenesis [31]. Spatial colocalization of THAP10‐expressing Tregs with inflammatory infiltrates in tumor‐adjacent regions offers preliminary evidence for immune dysregulation in AR–NPC relationships [32, 33], and CAPN14′s preferential expression at epithelial‐immune interfaces aligns with a hypothesized epithelial barrier dysfunction mechanism [34]. These observations are hypothesis‐generating, not definitive, which cautions against overinterpreting correlative findings in inflammation–cancer research.

Cellular heterogeneity revealed through single‐cell analysis—including distinct epithelial, immune, and stromal subpopulations—provides context for understanding how machine learning identifies discriminative patterns [35]. This integration illustrates how computational approaches can detect subtle cellular state transitions during progression from allergic inflammation to malignancy. However, the clinical significance of these transitions, including the proposed role of innate‐like B cells in chemotherapy response, remains unproven and requires experimental validation [36].

Our MR analysis suggests a potential causal association between AR and NPC (OR = 1.42, 95% CI: 1.18–1.76, *p* = 0.0003), offering genetic evidence that chronic mucosal inflammation may contribute to carcinogenesis via the proposed pathways [37]. This inference, combined with machine learning–derived signatures, could inform future stratified screening protocols—but not current clinical practice. A single MR result is insufficient to justify clinical action without replication in diverse cohorts [38, 39].

Functional pathway analysis identified dysregulation in mitochondrial processes, extracellular matrix–receptor interactions, and Wnt/Notch cascades—potential mechanistic targets for predictive models [40]. This multilayered approach, combining genetic evidence, machine learning, and pathway analysis, offers a preliminary framework for exploring personalized risk stratification but falls short of “paradigm‐shifting” progress.

The performance of our machine learning models, paired with biological context, provides a foundation for future translational research, though clinical utility remains unproven. Claims that these algorithms could “serve as clinical decision‐support tools” or guide “companion diagnostics” are premature [41, 42]. Such tools require wet‐lab validation of biomarkers, prospective clinical trials, and demonstration of improved patient outcomes—steps not yet undertaken here [24, 25].

Furthermore, candidate biomarkers (CAPN14 and THAP10) offer directions for future research into NPC risk stratification and therapeutic targeting, but their clinical value is unconfirmed. We emphasize these findings as starting points for hypothesis‐driven investigation, not evidence for clinical implementation.

Looking forward, our computational framework provides a template for exploring causal relationships between chronic inflammation and malignancy. Integrating MR with machine learning illustrates how population genetics might inform individual predictions, opening avenues for precision medicine research [28, 29]. However, functional validation is critical to confirm causality. Future work should expand to larger, diverse populations and integrate additional omics (e.g., proteomics and metabolomics) to enhance accuracy and mechanistic understanding [30].

## 5. Limitations

While single‐cell transcriptomics offers high‐resolution insights, this study is limited by sample size and the challenge of translating computational findings to clinical settings. As a computational discovery study, it lacks experimental validation (e.g., qPCR and IHC) of candidate biomarkers. Future research should validate findings in larger cohorts, explore pathway functionality via in vitro/in vivo models (e.g., qPCR validation of CAPN14/THAP10 in NPC tissues and CRISPR knockout assays), and integrate multiomics data to deepen mechanistic understanding.

## 6. Conclusion

In summary, this work provides a foundation for further inquiry into AR′s role in NPC development, with a novel analytical approach bridging genetic epidemiology and single‐cell biology. Its strength lies in generating testable hypotheses, which future studies must validate through expanded cohorts and experimental models.

## Ethics Statement

Not available. This study used publicly available summary data for Mendelian randomization analysis, which does not require ethics committee approval.

## Consent

The authors have nothing to report.

## Disclosure

All authors reviewed and approved the final manuscript.

## Conflicts of Interest

The authors declare no conflicts of interest.

## Author Contributions

Minqi Chen conceptualized the study, performed Mendelian randomization analysis, and drafted the manuscript. Bo Yang conducted single‐cell RNA sequencing and bioinformatic analysis. Changming Gong collected clinical samples and performed laboratory experiments. Xiao Liao assisted with statistical analysis and pathway enrichment. Kunwu He provided clinical expertise and critically revised the manuscript.

## Funding

No funding was received for this manuscript.

## Supporting information


**Supporting Information** Additional supporting information can be found online in the Supporting Information section. Table S1 provides comprehensive details for all instrumental SNPs.

## Data Availability

The data can be obtained from the corresponding author.
